# Systematic analysis of the expression profile and prognostic significance of m6A regulators and PD-L1 in hepatocellular carcinoma

**DOI:** 10.1007/s12672-022-00595-x

**Published:** 2022-11-25

**Authors:** Fanhua Kong, Kunpeng Wang, Liezhi Wang

**Affiliations:** 1 Zhongnan Hospital of Wuhan University, Institute of Hepatobiliary Diseases of Wuhan University, Transplant Center of Wuhan University, National Quality Control Center for Donated Organ Procurement, Hubei Key Laboratory of Medical Technology on Transplantation, Hubei Clinical Research Center for Natural Polymer Biological Liver, Hubei Engineering Center of Natural Polymer-based Medical Materials, Wuhan, 430071 Hubei China; 2 Department of General Surgery, Taizhou Central Hospital (Taizhou University Hospital), Taizhou, 318000 ZheJiang China

**Keywords:** Hepatocellular carcinoma (HCC), N6-methyladenosine (m6A), Bioinformatics analysis, Biomarker, Prognostic value, Immunology

## Abstract

**Background:**

Hepatocellular carcinoma (HCC) is a malignant tumor with poor prognosis. N6-methyladenosine (m6A) modification has dual biological functions in RNA modification and plays an important role in HCC.

**Methods:**

The GEO, TCGA, ONCOMINE, UALCAN, GEPIA, Kaplan–Meier plotter, cBioPortal for Cancer Genomics, STRING and TIMER2 databases were used for bioinformatic analyses. Quantitative polymerase chain reaction and western blotting were used to detect the expression of m6A regulators in HCC tissues.

**Results:**

The transcription of m6A regulators was upregulated in patients with HCC, and overexpression of YTHDF1/2, YTHDC1, RBM15 and METTL3 was significantly correlated with clinical stages of HCC. In addition, downregulation of ZC3H13 and METTL14 and upregulation of other m6A regulators were associated with a poor prognosis. A high mutation rate (89%) of m6A regulators was also observed in patients with HCC, and mutations in methylation regulators were associated with poor overall survival and disease-free survival. Finally, the expression of the YTHDF family was significantly associated with immune infiltration in the HCC microenvironment.

**Conclusion:**

m6A regulators and programmed death-ligand 1 may play an important role in the tumorigenesis and immune invasion and escape of HCC and may be risk factors affecting the survival of patients with HCC.

**Supplementary Information:**

The online version contains supplementary material available at 10.1007/s12672-022-00595-x.

## Introduction

Hepatocellular carcinoma (HCC) is a malignant tumour with poor prognosis [1, 2], with Asia and Africa having the highest incidence rates [3, 4]. However, biomarkers for early detection and prognostication of HCC and for the prediction and monitoring of treatment responses should be identified [5, 6]. In recent years, better immunotherapy outcomes have been observed in patients with advanced HCC owing to its safety and fewer adverse events. Therefore, immunotherapy can be considered a new strategy for clinical management. At present, there are still limitations in immunotherapy, and the application of immunotherapy in HCC is still limited. Studies have shown that immune escape of tumour cells plays an important role in the occurrence of HCC [7]. Therefore, further study of immune escape mechanism in HCC tumour microenvironment (TIME) is conducive to early detection of markers that can accurately predict prognosis.

N6-methyladenosine (m6A) is a major form of ribonucleic acid (RNA) modification, which can regulate the biological process of various cells and play an important role in the occurrence and development of diseases [8, 9]. The modification of m6A has a dual regulatory role, and its regulatory mechanism is mainly mediated by methyltransferase and demethylase [9, 10]. It can be identified according to different reader proteins and performed based on its unique biological functions [11]. The biological effects of m6A are mainly mediated by writer, eraser and reader proteins. The modification of m6A can be either methylated by the “writers” or demethylated by the “erasers” [12]. In addition to writers and erasers, another group known as readers can determine m6A modifications and perform different biological functions [13]. ‘Writers’ mainly include KIAA1429, METTL3, RBM15, ZC3H13, METTL16 and METTL14 and their cofactor WTAP [14]. ‘Readers’ mainly include HNRNPC, YTHDC1, YTHDC2, YTHDF1, YTHDF2 and YTHDF3 [15]. ‘Erasers’ primarily include ALKBH5 and FTO [9]. Studies have shown that m6A modification abnormalities play an important role in a variety of diseases, especially in tumours, where m6A modification abnormalities participate in the occurrence and development of tumours [16].

To date, the mechanism of immune escape from HCC remains limited. Programmed death receptor 1 (PD-1/PDCD1)/ programmed death ligand 1 (PD-L1) is considered to be the main cause promoting HCC immune escape, however, more evidence is still needed to confirm. PDCD1 is an immunosuppressive receptor that is expressed on the surface of immune cells, while PD-L1 is expressed in some tumour cells. Thus inducing immunosuppression and immune escape [17].

Although the study of m6A modification in HCC has made gratifying progress, its mechanism in tumour immune escape is still unknown. For example, YTHDF1 has been shown to play an important role in tumour immune escape by regulating the levels of CD8+ T cells and NK cells, thereby affecting the efficacy of tumour immunotherapy [18]. Therefore, m6A regulatory factors involved in the tumour immune response pathway may be a promising target for enhancing the clinical immunotherapeutic response.

Here, we used bioinformatics methods to investigate the relationship between m6A and PD-L1 and HCC prognosis, mutation, tumour stage, and immune cell infiltration. Our results suggest that the abnormal expression of m6A modifiers is associated with tumour stage and prognosis of HCC patients, and regulates immune escape from HCC.

## Materials and methods

### Datasets and preprocessing

We download the HCC RNA-seq transcriptome datasets (including 50 normal tissues and 374 samples of HCC) from TCGA database (https://portal.gdc.cancer.gov/). In addition, HCC datasets (GSE10143) were downloaded from the Gene Expression Omnibus (GEO) (www. ncbi.nlm.nih. gov/ geo/). These data sets included survival time, survival status, age, gender, grade, stage and lymph node metastasis classification. The GEO and TCGA cohort were analyzed using the Perl programming language (version Strawberry-Perl-5.30.0; https://www.perl.org) [19]. In addition, we also used Perl to process and analyze clinical data to obtain complete pathological information of clinical samples.

### Identification of differentially expressed m6A regulators and prognosis of HCC

We analyzed the differences in the expression of m6A regulators between HCC and normal tissues using the packages of "limma" and "reshape2". The Overall survival (OS) were assessed by the “survival” and “survminer” packages.

### Correlations of m6A regulators with tumor microenvironment in HCC

The ESTIMATE algorithm was used to evaluate the immune score, and the CIBERSORT algorithm [20] was used to analyze the 22 immune cell subsets in HCC. The abundance of 22 infiltrating immune cells in heterogeneous samples was quantified using CIBERSORT.

### ONCOMINE (http://www.oncomine.org)

The ONCOMINE database was used to analyse the transcription levels of m6A RNA methylation and PD-L1 in HCC tumour tissues and the corresponding adjacent healthy control tissues. The cell colour was determined by the best gene rank percentile for analysis within the cell, and a Q–Q graph and histogram were used to detect whether the sample data followed a normal distribution. Subsequently, the Student’s t-test was used to generate p-values. A p-value of 0.05, a fold change of 2 and a gene rank in the top 10% were set as the thresholds of significance.

### UALCAN (http://ualcan.path.uab.edu/)

UALCAN is a clinical database that is used to analyse gene expression among tumour subgroups, survival and cancer genome-based mapping (TCGA) of level 3 RNA-seq data across 31 cancer types [21]. In this study, UALCAN was used to examine the distinct expression levels of tumour and healthy tissues. TCGA database was used for analysing the expression of m6A regulatory factors and PD-L1 in HCC, and the data were downloaded. The Student’s t-test was used to generate p-values, with a p-value cutoff of 0.05.

### GEPIA (http://gepia.cancer-pku.cn/)

GEPIA is a web server for cancer and normal gene expression profiling and interaction analyses [22]. In this study, the ‘Multiple Gene Comparison’ module was used to evaluate the expression of multiple m6A regulators. The ‘Expression DIY’ module was used to examine the relationship between 15 m6A regulators and clinicopathological parameters. The relationship between the expression of m6A regulators and tumour stages was analysed using the ‘Single Gene Analysis’ module. The Student’s *t*-test was used to generate *p*-values, with a *p*-value cutoff of 0.05.

### Kaplan–Meier Plotter (http://kmplot.com/analysis/)

The Kaplan–Meier (K–M) plotter can be used to assess the effects of approximately 54,000 genes on survival across 21 cancer types [23]. In this study, K-M curves were used to analyse the relationship between gene mutations in m6A regulators and PD-L1 and overall survival (OS), progression-free survival (PFS), recurrence-free survival (RFS) and disease-specific survival (DSS) in patients with HCC. The log-rank test was used to evaluate significant differences in the survival curves. A *p*-value of < *0.05* was considered statistically significant. In addition, the K-M plotter was used to evaluate the prognostic value of m6A regulators and PD-L1 mRNA expression; patients with HCC were divided into the high- and low-expression groups based on the median values of mRNA expression, and the results were validated using K-M survival curves based on the hazard ratio (HR) with 95% confidence intervals (CIs) and log-rank *p*-values. Patients were grouped to select the best cutoff, and survival analysis was performed, including OS, FP and PPS analyses. A *p*-value of < 0.05 was considered statistically significant.

### TCGA Data and cBioPortal for Cancer Genomics (http://www.cbioportal.org)

TCGA database was used to analyse the genomic profiles of m6A regulators and PD-L1, including mutations, putative copy number alterations from GISTIC and mRNA expression z-scores (RNA-Seq V2 RSEM) with a z-score threshold of ± 1.8. The co-expression of m6A regulators and PD-L1 was analysed using the ‘co-expression’ module of cBioPortal. Pearson correlation coefficient was used to examine the correlation among co-expressed genes of m6A regulators and PD-L1. The top 10 co-expressed genes with the highest Pearson correlation coefficients of each m6A RNA methylation regulator and PD-L1 were screened. In the cBioPortal database, ‘Liver Hepatocellular Carcinoma (TCGA, Firehose Legacy)’ was selected in the Liver section, and the ‘Query By Gene’ function was used for analysis. The ‘mRNA expression z-scores relative to diploid samples (RNA Seq V2 RSEM)’ and ‘Samples with mRNA data (RNA Seq V2)’ modules were selected for genomic profiling. The correlation between the expression of m6A regulators and PD-L1 was analysed using the ‘Mutual Exclusivity’ module.

### STRING (http://www.string-db.org)

STRING is a database of known and predicted protein–protein interactions (PPIs)[24]. In this study, STRING was used to analyse PPI networks of m6A regulators and PD-L1. *Homo sapiens* was selected as the species, and a combined score of > 0.7 was considered statistically significant. The nodes indicated proteins, the edges indicated the interaction of proteins and disconnected nodes in the network were hidden.

### DAVID (https://david.ncifcrf.gov/summary.jsp)

Gene Ontology (GO) and Kyoto Encyclopedia of Genes and Genomes (KEGG) analyses were used to examine the functions of mutated m6A regulators and 170 genes significantly related to the mutations of m6A regulators using the DAVID database. GO analysis was used to predict the functions of mutated m6A regulators and 170 genes significantly related to the mutations of m6A regulators, whereas KEGG analysis was used to predict the related pathways of mutated m6A regulators and 170 co-expressed genes significantly related to the mutations of m6A regulators. A p-value of < 0.05 was considered statistically significant.

### TIMER2 (http://timer.cistrome.org/)

The relationship between the expression of the YTHDF gene family and immune infiltration across all TCGA tumour types was explored using the ‘Immune Genes’ module of the TIMER2 Web server. Immune cells such as cancer-associated fibroblasts and regulatory T cells (Tregs) were selected. The TIMER, CIBERSORT, CIBERSORT-ABS, QUANTISEQ, XCELL, MCPCOUNTER, TIDE and EPIC algorithms were used for assessing immune infiltration. p-values and partial correlation (cor) values were evaluated using the purity-adjusted Spearman’s rank correlation test. The data were visualised on a heatmap and scatter plot.

### RNA extraction and qRT-PCR

Tumour and para-cancerous tissues were treated with the TRIzol reagent (Invitrogen, USA) for 10 min and centrifuged at 12,000*g* at 4 °C for 15 min. Thereafter, RNA to be suppressed was collected and mixed with isopropanol for its isolation. After RNA was obtained, its purity and concentration were analysed using a NanoDrop 1000 spectrophotometer (Thermo Fisher, USA). cDNA was synthesised using a high-capacity cDNA reverse transcription kit (Life Tec, America). The primers used for genes are listed in Table S1. qRT-PCR was performed using 2X Universal SYBR Green Fast qPCR mix (Abclonal, China) on a LightCycler 96 system (Roche, America). All experiments were performed in triplicate.

### Western blotting (WB)

HCC specimens were collected during surgery. The proteins separated on gels after electrophoresis are transferred to PVDF membranes and subsequently incubated with primary and secondary antibodies. The following primary antibodies were incubated for 12 h at 4 °C: GAPDH (1:10,000, ABclonal), YTHDF1 (1:1000, ABclonal), YTHDF2 (1:1000, ABclonal) and YTHDF3 (1:1000, ABclonal), KIAA1429(1:1000, Proteintech), METTL3(1:1000, Proteintech), RBM15(1:1000, Proteintech), ZC3H13(1:1000, Proteintech), METTL16(1:1000, Proteintech), ALKBH5(1:1000, Proteintech), METTL14(1:1000, Proteintech), WTAP(1:1000, Proteintech), HNRNPC(1:1000, Proteintech), YTHDC1(1:1000, Proteintech), YTHDC2(1:1000, Proteintech), FTO(1:1000, Proteintech). The secondary antibody (1:5000, ABclonal) was incubated for 2 h at room temperature. Immunoreactive bands were developed using an enhanced chemiluminescence detection kit (Genview Scientific Inc., USA). All experiments were performed in triplicate.

### RNA m6A quantification

A total of 20 pairs of surgical specimens (tumour and para-cancerous tissues) were collected from patients with HCC. Total RNA was extracted using the TRIzol reagent (Invitrogen, CA, USA) as described above, and its purity and concentration were analysed using a NanoDrop 1000 spectrophotometer (Thermo Fisher Scientific, Waltham, MA, USA). The m6A modification level of total RNA was examined using the EpiQuik m6A RNA Methylation Quantification Kit (p-9005; Epigentek Group Inc., Farmingdale, NY, USA) according to the manufacturer’s instructions. Briefly, 200 ng of RNA along with the m6A standard were coated on assay wells, followed by the addition of capture antibody and detection antibody solutions. m6A levels were quantified colourimetrically by reading the absorbance of each well at a wavelength of 450 nm (OD450), and subsequent evaluation was performed based on the standard curve.

### Ethics statement

This study was approved by the Medical Ethics Committee of Taizhou Central Hospital (Approval No.: 2020L-12-25). All experiments were conducted following the study protocol and the research was carried out following the guidelines of the ethics committee listed in the ethics statement.

### Statistical analysis

All descriptive data were expressed as mean ± standard deviation. Statistical analyses were performed using the SPSS Statistics version 21.0 (IBM, USA) software. Paired t-test was used to analyse differences between two groups. A *p*-value of < 0.05 indicated significant differences.

## Results

### Abnormal expression of m6A regulators is associated with tumour stages in patients with HCC

**Fig. 1:**
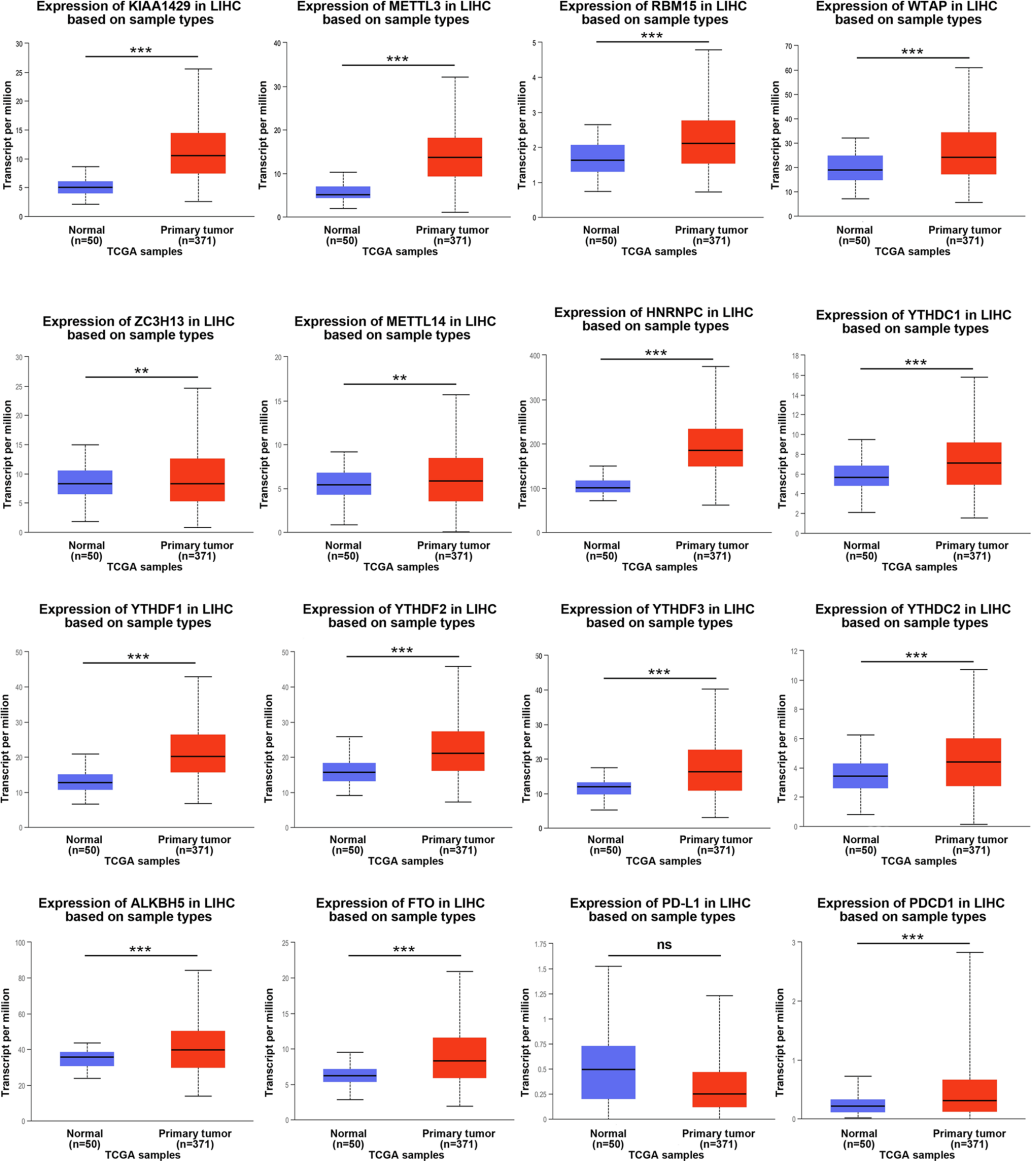
mRNA expression of m6A regulators in HCC tumor tissues and adjacent liver tissues. Compared with normal liver tissues, the expression of m6A regulators in HCC tissues was higher than that in normal liver tissues. ***p < 0.001, **p < *0.01*

**Fig. 2:**
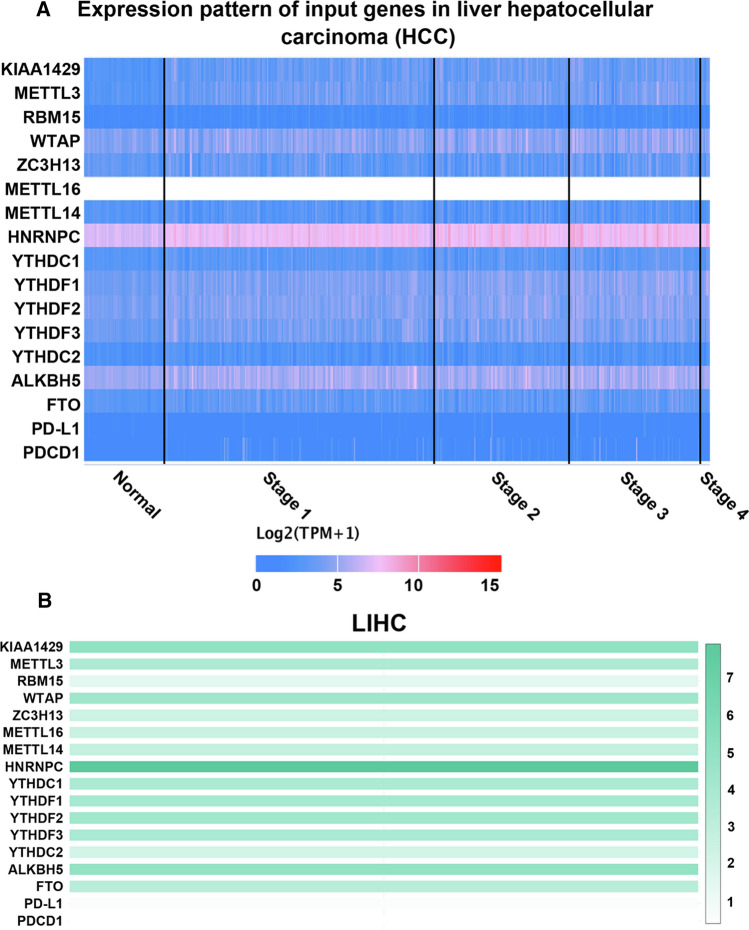
**A** Differential heat maps of expression of m6A regulators and PD-L1 in HCC tumor tissues (tumor stage) and para-cancerous tissues. **B** Relative levels of m6A regulators and PD-L1 in HCC. The expression of HNRNPC was the highest in HCC

The expression of m6A regulators and PD-L1 in HCC was analysed using ONCOMINE and UALCAN. A total of 50 healthy tissues and 371 HCC tissues were examined using the UALCAN database. As shown in Fig. 1, the expression of all genes, except PD-L1, was significantly upregulated in HCC tissues. In addition, in the ONCOMINE database, analysis of multiple HCC datasets showed that the expression of m6A regulators was significantly higher in HCC tissues than in healthy tissues (Table S2), suggesting that m6A regulators play an important role in the occurrence and development of HCC and may serve as molecular markers for early diagnosis and prognosis of HCC. Furthermore, the relative expression levels of each m6A RNA methylation regulator and PD-L1 were compared in HCC tissues. As shown in Fig. 2 and Figure S1A, m6A regulators were generally significantly upregulated in HCC tissues.

**Fig. 3:**
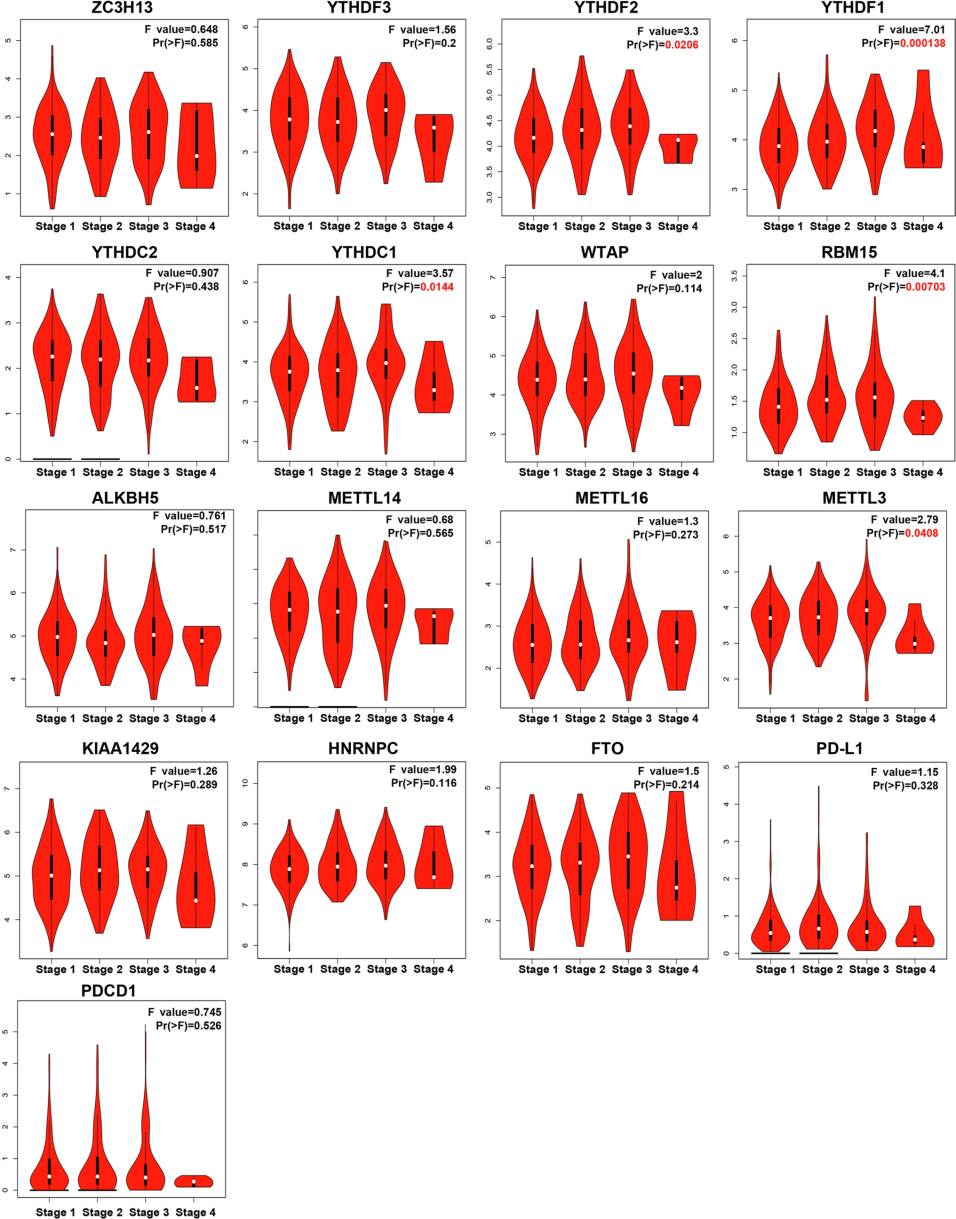
Correlation between expression of m6A regulators and tumor staging (GEPIA) in patients with HCC. The mRNA expressions of YTHDF1/2, YTHDC1, METTL3 and RBM15 were significantly correlated with individual tumor stage

Furthermore, the relationship between the expression of m6A regulators and clinical stages of HCC was evaluated using the GEPIA database. As shown in Fig. 3, the expression of YTHDF1, YTHDF2, YTHDC1, RBM15 and METTL3 was found to be significantly associated with HCC staging, whereas other genes did not have a significant relationship with the clinical stages of HCC. Moreover, the expression of m6A methylation regulators was downregulated in the late stage of HCC (stage 4), suggesting that m6A modification mainly occurs in the early stages of HCC. This finding may also be related to the small sample size of patients with stage 4 disease; therefore, the sample size should be expanded for further verification. In conclusion, the abovementioned results suggest that the expression level of m6A methylation regulators is significantly correlated with individual tumour stage and is higher in patients with early-stage disease.

### Prognostic characteristics of m6A regulators in patients with HCC

**Fig. 4:**
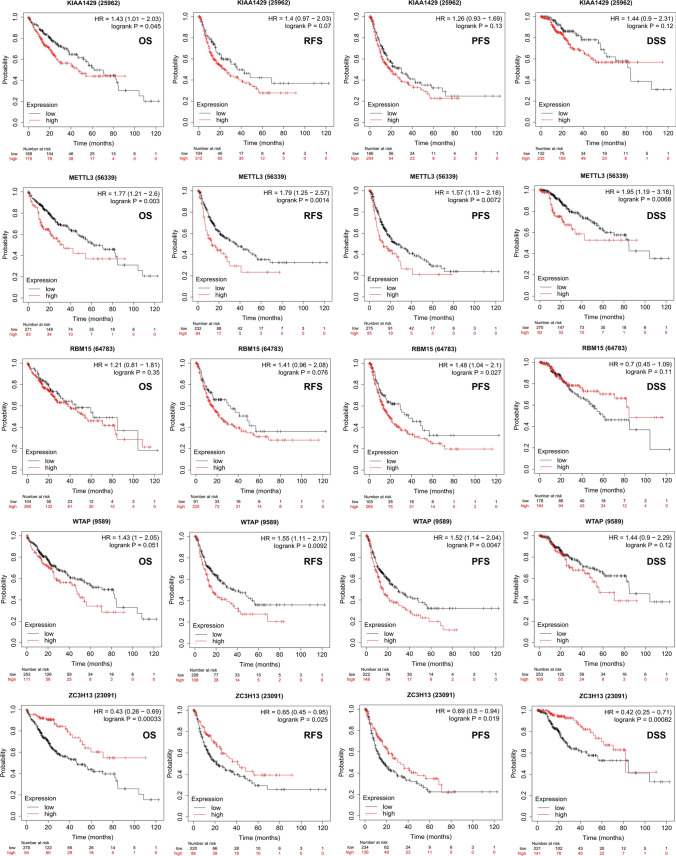
Prognostic feature of mRNA expression of distinct m6A regulators and PD-L1 in HCC patients (Kaplan–Meier plotter). The OS, RFS, PFS and DSS survival curves comparing patients with high (red) and low (black) m6A regulators and PD-L1 expression in HCC were plotted using the Kaplan–Meier plotter database at the threshold of p-value of < 0.05

**Fig. 5:**
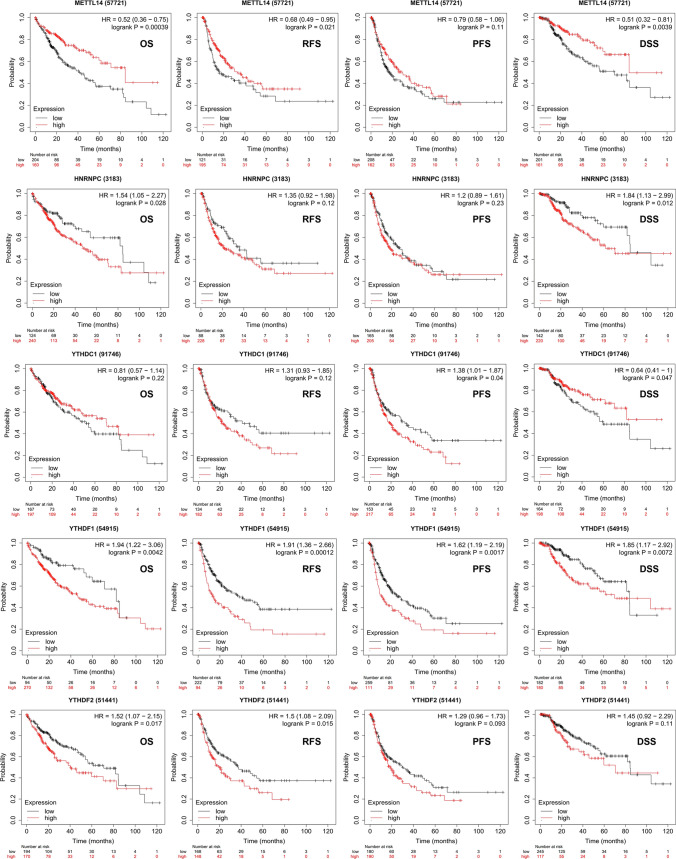
Prognostic feature of mRNA expression of distinct m6A regulators and PD-L1 in HCC patients (Kaplan–Meier plotter). The OS, RFS, PFS and DSS survival curves comparing patients with high (red) and low (black) m6A regulators and PD-L1 expression in HCC were plotted using the Kaplan–Meier plotter database at the threshold of p-value of < 0.05

To investigate the effects of m6A regulators on the prognosis of HCC, the relationship between the expression of m6A regulators and PD-L1 and OS, relapse-free survival (RFS), PFS and DSS was examined using the K-M plotter. As shown in Table S3, patients in each cohort were divided into the low- and high-risk groups based on the cut-off values. The cut-off values for OS associated with m6A regulators are shown in Figure S2. As shown in Fig. 4, upregulated mRNA expression of KIAA1429 was associated with poor OS (*p* = *0.045*) but was not associated with RFS, PFS or DSS. Upregulated mRNA expression of METTL3 was associated with poor OS (*p* = *0.003*), RFS (*p* = *0.0014*), PFS (*p* = *0.0072*) and DSS (*p* = *0.0068*), suggesting that patients with HCC with upregulated METTL3 expression had a poor prognosis. In addition, upregulated mRNA expression of RBM15 was associated with PFS (*p* = *0.02*7), and upregulated mRNA expression of WTAP was correlated with RFS (*p* = *0.0092*) and PFS (*p* = *0.0047*). Furthermore, downregulated mRNA expression of ZC3H13 was associated with poor OS (*p* = *0.00033*), RFS (*p* = *0.025*), PFS (*p* = *0.019*) and DSS (*p* = *0.00082*), whereas downregulated mRNA expression of METTL14 was associated with poor OS (*p* = *0.00039*), RFS (*p* = *0.021*) and DSS (*p* = *0.0039*) (Fig. 5). Low expression of ZC3H13 and METTL14 as m6A ‘writer’ genes has been associated with a poor prognosis in breast and colon cancers [25–27] and may lead to downregulation of m6A RNA modification in tumours, thereby reducing the level of immune cell infiltration and resulting in a poor prognosis, which is consistent with the results of this study. Upregulated mRNA expression of HNRNPC was associated with poor OS (*p* = *0.028*) and DSS (*p* = *0.012*) (Fig. 5). Upregulated mRNA expression of YTHDC1 was associated with poor PFS (*p* = *0.04*) and good DSS (*p* = *0.047*). In addition, upregulated mRNA expression of YTHDF1 was associated with poor OS (*p* = *0.0042*), RFS (*p* = *0.00012*), PFS (*p* = *0.0017*) and DSS (*p* = *0.0072*), whereas mRNA expression of YTHDF2 was associated with poor OS (*p* = *0.017*) and RFS (*p* = *0.015*). Other m6A regulators associated with the prognosis of HCC are shown in Figure S3 and Figure S4. In conclusion, abnormal expression of m6A regulators plays an important regulatory role in the prognosis of HCC, with clinical significance. For example, upregulated expression of METTL3, WTAP, YTHDF1 and YTHDF2 indicates a poor prognosis of HCC. However, low expression of ZC3H13 and METTL14 as tumour suppressor genes can also lead to a poor prognosis of HCC, indicating that m6A regulators play an extremely important role in the prognosis of HCC.

### Mutations in m6A regulators and their relationship with survival in patients with HCC

**Fig. 6:**
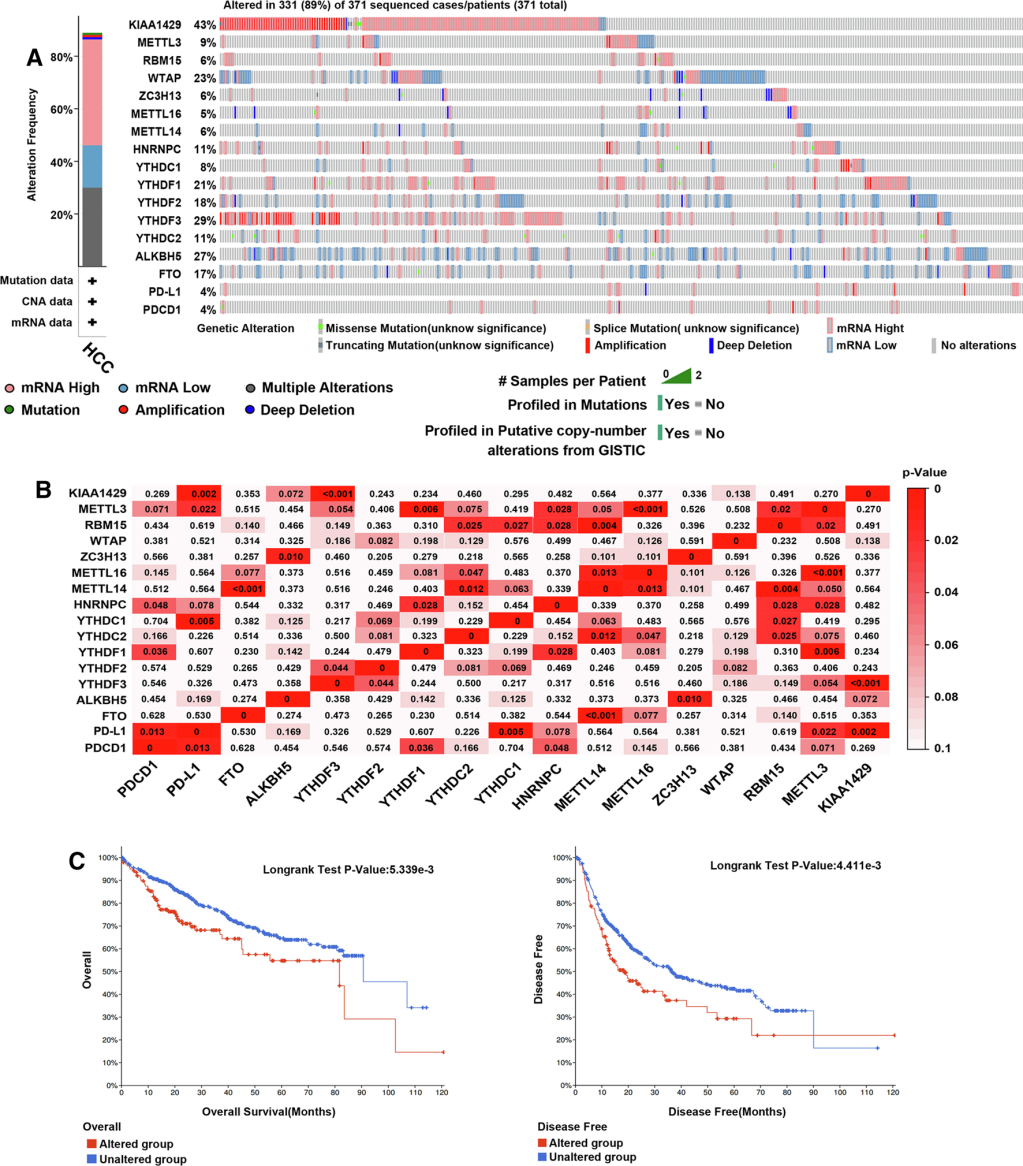
Genetic mutations in m6A regulators, PD-L1 and their association with OS and DFS of HCC patients (cBioPortal). **A** Summary of alterations in different expressed m6A regulators and PD-L1 in HCC. **B** Correlations of different m6A regulators and PD-L1 with each other in HCC. **C** Genetic alterations in m6A regulators and PD-L1 were related to shorter OS and DFS of HCC patients

Epigenetic changes play a crucial role in the occurrence and development of tumours. The effects of mutated m6A regulators on the progression and prognosis of HCC remain unknown. In this study, mutations in m6A regulators in patients with HCC were analysed using the online tool cBioPortal for Cancer Genomics (TCGA, Firehose Legacy). Mutated m6A regulators were found in 331 (89%) of the 371 patients with HCC (Fig. 6A). Among these regulators, the mutation rates of KIAA1429, YTHDF3, ALKBH5, WTAP, YTHDF1, YTHDF2 and FTO were 43%, 29%, 27%, 23%, 21%, 18% and 17%, respectively. By analyzing the GEO and TCGA databases, we found that m6A modification also had gene mutations (Figure S1B). In addition, the mRNA expression of these regulators and PD-L1 (RNA Seq V2 RSEM) in LIHC (TCGA, Firehose Legacy) was analysed using cBioPortal, followed by Pearson correlation analysis. The correlation between RNA methylation regulators and PD-L1 was observed (Fig. 6B), and the results showed that KIAA1429 was positively correlated with PD-L1 and YTHDF3; METTL3 was positively correlated with PD-L1, YTHDF1, HNRNPC, METTL14, MTEEL16 and RBM15; RBM15 was positively correlated with YTHDC2, YTHDC1, HNRNPC and METTL14; ZC3H13 was positively correlated with ALKBH5; METTL16 was positively correlated with YTHDC2 and METTL14; METTL14 was positively correlated with FTO and YTHDC2 and HNRNPC was positively correlated with PDCD1 and YTHDF1. In addition, YTHDC1 was positively correlated with PD-L1; YTHDF1 was positively correlated with PDCD1; YTHDF2 was positively correlated with YTHDF3 and PD-L1 was positively correlated with PDCD1. In addition, the relationship between mutated m6A regulators and OS and disease-free survival (DFS) in patients with HCC was investigated. The K-M plot and log-rank test results showed that mutated m6A regulators were associated with shorter OS (Fig. 6C,  *p* = 5.339E−3) and DFS (Fig. 6C,  *p* = 4.411E−3) in patients with HCC. These results suggest that mutations in m6A regulators and the PD-L1 gene play an important role in the prognosis of HCC.

### GO and KEGG enrichment analyses of m6A regulators, PD-L1 and 170 co-expressed genes in patients with HCC

**Fig. 7:**
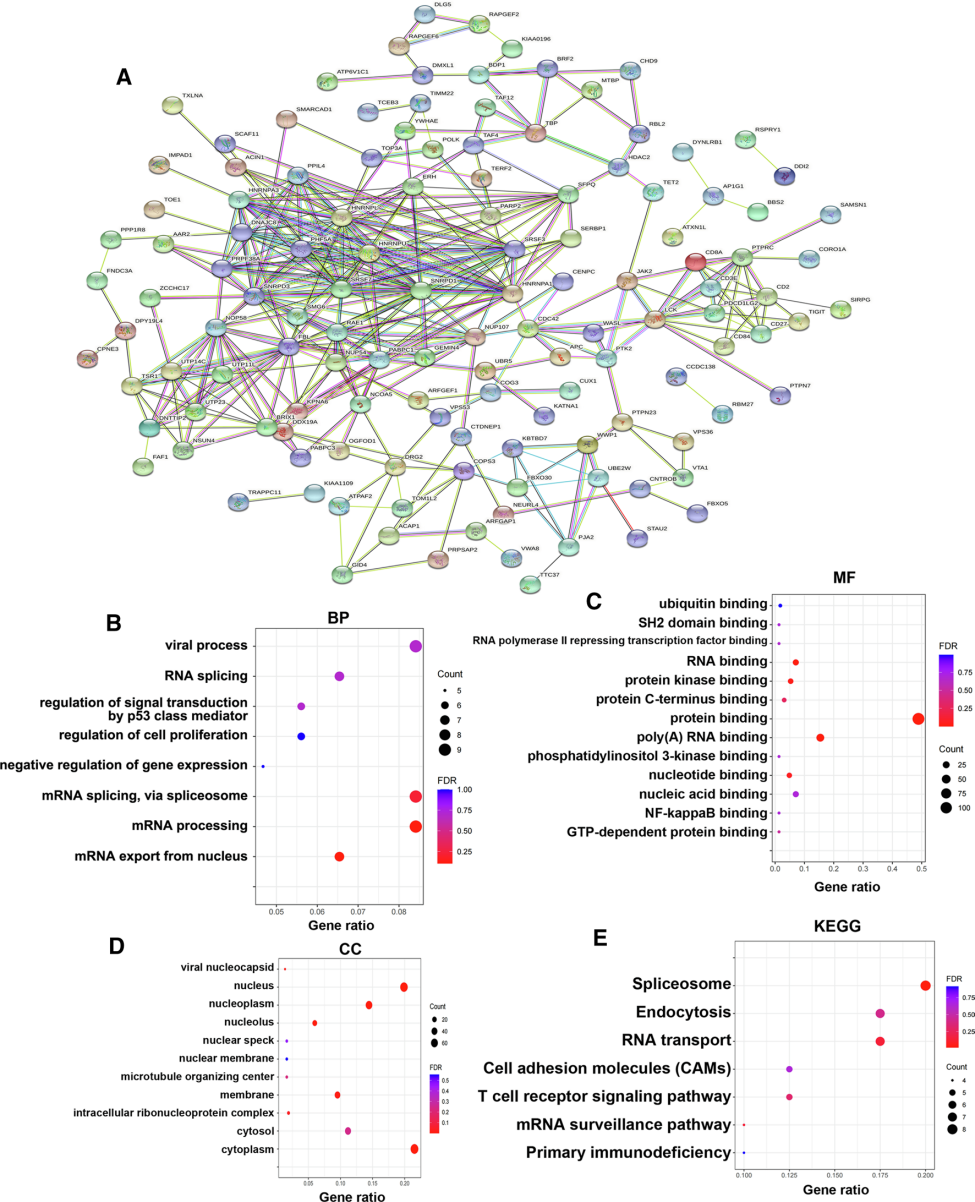
GO and KEGG enrichment analysis of m6A regulators and their 170 co-expression genes in HCC patients. (STRING and DAVID). **A** PPI network. The nodes meant proteins; the edges meant the interaction of proteins **B** BP. **C** MF. **D** CC. **E** KEGG

After analysing the gene mutations of m6A regulators and their prognostic value in patients with HCC, the ‘co-expression’ module of cBioPortal was used to analyse the top 10 co-expressed genes (among 170 genes) significantly associated with the mutations of each m6A RNA methylation regulator (see Table S4 for specific co-expressed genes). Subsequently, a PPI network was established using the STRING database. As shown in Fig. 7A, the HNRNP family (HNRNPA1/3, HNRNPL and HNRNPU), SNRPD1/3, SRSF3/7, PHF5A and PRPF38A were found to be closely associated with mutated m6A regulators. The HNRNP family is a class of RNA-binding proteins with various key cellular functions. Cell dysfunction, including selective splicing, translation and RNA processing, plays an important role in tumorigenesis. Therefore, the HNRNP family has attracted increasing attention owing to its association with cancer progression. In addition, SNRPD1/3 plays a regulatory role in breast cancer through cell cycle regulation, and SRSF3/7 is an RNA-binding protein associated with metastasis and recurrence. PHF5A and PRPF38A play an important role in regulating tumour proliferation and migration. Therefore, in this study, GO and KEGG analyses were performed using DAVID to analyse the potential role of m6A regulators and their 170 co-expressed genes in HCC (Table S5). As shown in Fig. 7B–E, mutated m6A regulators were found to significantly regulate biological processes (BPs), such as regulation of signal transduction by a p53 class mediator, cell proliferation, T-cell co-stimulation, mRNA processing and positive regulation of T-cell proliferation (Fig. 7B). Furthermore, molecular functions included protein, poly(A) RNA and protein kinase binding (Fig. 7C), whereas cellular components included the cytoplasm, nucleus and nucleoplasm (Fig. 7D). KEGG analysis indicated that mutated m6A regulators were associated with the activation of the T-cell receptor signalling pathway, RNA transport and spliceosomes (Fig. 7E). Moreover, the relationship between mutated m6A regulators and the clinical characteristics of patients with HCC was also analysed (Figure S5, Table S6). The results suggested that mutated m6A regulators were associated with vascular invasion. In addition, the cancer type, neohistological grade and primary tumour site were significantly correlated with the mutated regulators, suggesting that the regulators were associated with a poor prognosis.

### Immune infiltration analysis of m6A regulators in the tumour microenvironment of patients with HCC

**Fig. 8:**
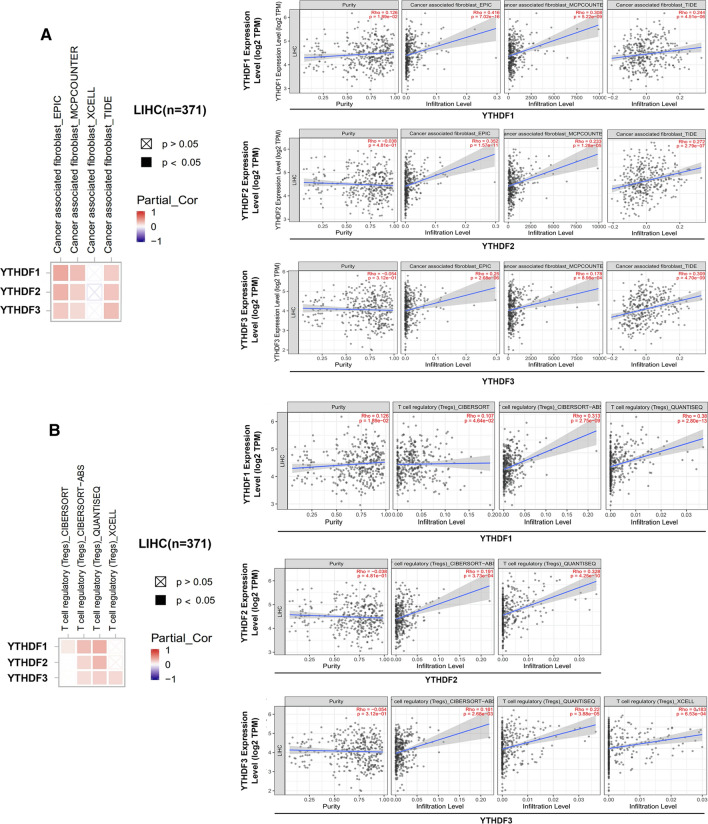
Correlation analysis between YTHDF family expression and immune infiltration of cancer-associated fibroblasts and Regulatory T cells (Treg). Different algorithms were used to explore the potential correlation between the expression level of the YTHDF family gene and the infiltration level of cancer-associated fibroblasts across and Tregs of HCC in TCGA

Tumour-infiltrating immune cells, as an important part of the tumour microenvironment, are closely related to the occurrence, progression or metastasis of tumours. Previous studies have shown that differences in tumour tissue expression, survival and gene mutations were highly significant among genes in the YTHDF family. In addition, studies have shown that m6A regulators play an important role in the regulation of tumour immunity. Therefore, the underlying mechanisms and role of the YTHDF family in immune infiltration within the tumour microenvironment should be further investigated. In this study, the CIBERSORT, CIBERSORT-ABS, QUANTISEQ, XCELL, MCPCOUNTER, TIDE and EPIC algorithms were used to investigate the potential relationship between the infiltration levels of various immune cells and the expression of the YTHDF family in TCGA-HCC cohort (371 patients). The role of the YTHDF family in the infiltration of tumour-associated fibroblasts and Tregs was determined using the MCPCOUNTER, TIDE and EPIC algorithms, which showed that the expression of the YTHDF family was significantly positively correlated with the infiltration of tumour-associated fibroblasts in HCC (Fig. 8A). In addition, analyses performed using the CIBERSORT, CIBERSORT-ABS, QUANTISEQ and XCELL algorithms (Fig. 8B) showed that the expression of the YTHDF family was significantly correlated with Treg infiltration in HCC, suggesting its important role in the regulation of tumour immunity and immune escape. In addition, analysis of data sets downloaded from GEO and TCGA databases revealed that YTHDF family was associated with infiltration of T cells, B cells, and macrophages (Figure S1C).

### qRT-PCR and WB validated the upregulation of m6A regulators in HCC

**Fig. 9:**
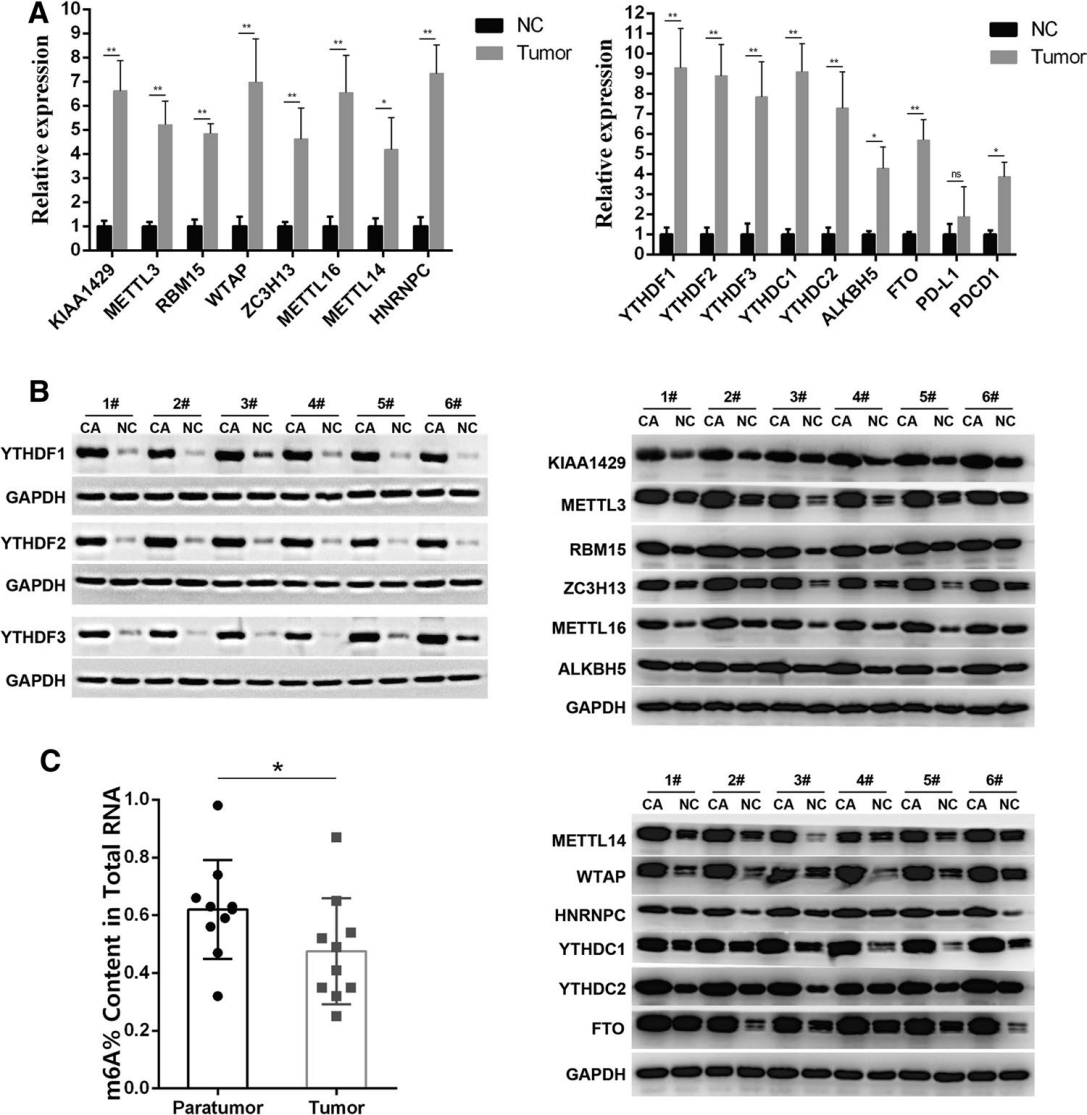
Expression of m6A regulators in HCC. **A** Verify the expression of m6A regulators in HCC patients by qRT-PCR. **B** Western blotting was used to verify the expression of m6A regulators in patients with HCC. **C** Global RNA m6A methylation of HCC was evaluated by the m6A RNA Methylation Quantification Kit. The m6A contents of total RNAs in tumor tissues(n = 10) paired with para-tumor tissues (n = 10)

Analyses performed using UALCAN, TCGA and other databases revealed that the expression of m6A regulators was upregulated in HCC. To further verify these results, surgical specimens were collected from 20 patients clinically and pathologically diagnosed with HCC. qRT-PCR and WB were used to verify changes in the expression of m6A regulators. As shown in Fig. 9A and Table S7, the results of qRT-PCR showed that the expression of m6A regulators was upregulated in HCC, and the results of WB (Fig. 9B) showed that the expression of m6A regulators were upregulated in HCC. These results are consistent with those mentioned above. In addition, to examine the potential role of m6A modification in HCC, the EpiQuik m6A RNA Methylation Quantification Kit was used to detect m6A modification levels in the total RNA of tumour and para-cancerous tissues. The results revealed that m6A modification levels were lower in HCC tissues than in the corresponding adjacent tissues (Fig. 9C). These results suggest that the overall m6A modification levels in HCC are mainly mediated by eraser and reader proteins, which warrants further study.

## Discussion

In this study, we found that the expression of m6A regulators was up-regulated in HCC tissues, and the up-regulated expression of YTHDF1/2, YTHDC1, RBM15 and METTL3 was significantly correlated with the tumour stage of HCC patients. In addition, the abnormal expression of m6A regulator reduced the OS, RFS, PFS and DSS of HCC patients, which was consistent with the results of previous studies. For example, upregulation of METTL3 is associated with poor prognosis in HCC patients [28]. In addition, YTHDF2 expression was up-regulated in PCA tissues and cell lines, and was closely related to the level of m6A in PCA tissues and cell lines [29]. FTO and YTHDF1 have also been shown to be associated with poor prognosis in HCC patients, and they affect the prognosis of HCC mainly by regulating the level of m6A modification [30]. Therefore, m6A regulators are potential biomarkers and potential therapeutic targets for HCC. Finally, we also found that the expression of PDCD1 was up-regulated in HCC tissues, while the expression of PD-L1 was not significantly different between HCC and healthy tissues. Survival analysis suggested that low expression of PDCD1 was associated with poor prognosis, whereas PD-L1 did not show a significant association [31]. Studies have shown that abnormal expression of PD-L1 in tumours promotes tumour immune escape, but the prognostic effect of PD-L1 in HCC remains inconsistent, with some studies reporting conflicting results. For example, Sideras et al. [32] reported that high expression of PD-L1 indicated significantly improved survival of HCC patients. Adaptive immune resistance has been used to explain this discrepancy. Upregulation of PD-L1 represents the presence of immune surveillance and may be associated with better prognosis. These results suggest that the two mechanisms may co-exist and that the main mechanism may change at different points in time depending on the immunogenicity of the tumour [33].

In recent years, oncogenic mechanisms underlying tumour prognosis have emerged as a prime focus area for research. In this study, upregulated expression of m6A regulatory factors, such as the YTHDF family, METTL3, RBM15, WTAP and KIAA1429, was found to be closely associated with a poor prognosis of HCC. In addition, low expression of ZC3H13 and METTL14 was associated with a poor prognosis. Gong et al. found that low expression of ZC3H13 and METTL14 was associated with a poor prognosis in breast and colon cancers [25–27], a finding consistent with that of the present study. In addition, Ma et al. [34] found that downregulation of METTL14 is associated with HCC metastasis and can be used as a prognostic factor for HCC, and METTL14 deletion can enhance the ability of HCC metastasis. Chen et al. [35] found that ALKBH5 was downregulated in HCC, and reduced ALKBH5 expression was an independent prognostic factor for identifying worsening survival of patients with HCC. This finding is different from that obtained using the ONCOMINE and UALCAN databases in this study. This inconsistency may be because HCC is mostly complicated by hepatitis B virus infection in Eastern countries. Moreover, the results of the two studies on FTO remain unclear; FTO may serve as an oncogene or a tumour suppressor gene in HCC. Previous studies have also indicated that the effects of FTO on the proliferation ability of different HCC cells are controversial [36, 37]. In addition, Wang et al. [38] demonstrated that the absence of METTL3 and METTL14 reduced the viability of HeLa cells. In this study, upregulation of METTL3 and downregulation of METTL14 were associated with the poor prognosis of HCC. Although METTL3, METTL14 and WTAP can form RNA methyltransferase complexes, which regulate the fate of tumour development, studies have shown that METTL3 and METTL14 also have independent regulatory functions in regulating transcription. For example, Liu et al. [39] established the function of the METTL3–MeTTL14 complex independent of m6A. The reassignment of METTL3 to the promoter of SASP gene and the reassignment of METTL14 to the enhancer of SASP gene suggest that the METTL3–METTL14 complex plays an important role in regulating transcription independent of its m6A function, thereby playing an independent regulatory role. Liu et al. demonstrated that METTL3 and METTL14 play a contradicting regulatory role in HCC, and their expression and prognostic value are also opposite, which indicates the synergistic role of METTL3 and METTL14 in the catalytic modification of m6A [40]. These studies indicate that there is a complex regulatory network of m6A modification in different tissues, cell lines and spatiotemporal models and also verify the dual and complex regulatory mechanisms of m6A modification in tumours. Therefore, although METTL14 and ALKBH5 play an important role in inhibiting HCC metastasis, they may regulate the progression of HCC through different functional mechanisms dependent on or independent of m6A modification, which requires further investigation and validation.

Epigenetic changes play an important role in early malignant tumours [41]. In this study, m6A regulators were found to have an extremely high mutation rate (89%) in HCC, and the high mutation rates were significantly associated with shorter OS and DFS. Studies have shown that the mutation of m6A regulatory factors is significantly correlated with clinicopathological characteristics, and that of TP53 is significantly associated with poor OS and DFS [42]. Zhu et al. found that the mutation and differential expression of m6A resulted in a significant relationship between increased m6A levels and poor survival, especially in patients with HCC with TP53 mutations [43]. Genetic alterations in the m6A gene may cooperate with TP53 and its regulatory targets in the pathogenesis of HCC [43]. This finding is consistent with that of GO and KEGG enrichment analyses in this study (activation of the P53 signal). Therefore, mutated m6A regulatory factors may serve as new clinical targets for HCC treatment.

From an epigenetic point of view, tissue specificity and uneven distribution of m6A modification provide a new direction for understanding the pathogenesis of different diseases, especially tumours. However, whether m6A modification is related to the tumour microenvironment has been rarely reported. The tumour microenvironment plays an important regulatory role in tumorigenesis, and its heterogeneity can reflect patient prognosis and treatment response [44]. Further investigation of the mechanisms of m6A methylation in tumour-infiltrating lymphocytes and evaluation of immune scores can help to promote clinical diagnosis and targeted tumour therapy. Li et al. [45] reported that METTL3 or METT14 deletion induces T-cell deproliferation and differentiation, thereby reducing IL-7 sensitivity in vivo. Han et al. [18] found that the infiltration levels of CD8+ T and NK cells were increased in mouse models of YTHDF1-deficient tumours, which enhanced the cross-expression of tumour antigens and cross-primers of CD8+ T cells in vivo. These studies suggest that m6A regulators are, to some extent, involved in time regulation and tumour immune cell infiltration. In this study, the most significant differences in the expression pattern, prognostic value and gene mutations were observed among the members of the YTHDF family in HCC. Therefore, the immune infiltration status of the YTHDF family in HCC was further analysed, which showed that the YTHDF family was significantly associated with tumour-associated fibroblasts and Tregs in the HCC tumour microenvironment, which may promote immune escape. Moreover, Tregs play an important role in regulating tumour immunotherapy and immune escape. Therefore, mechanisms underlying m6A regulator-mediated Treg immune escape in HCC should be investigated further.

In conclusion, the expression, prognostic value and immune invasion levels of m6A regulators in HCC were elucidated, and potential mechanisms of m6A regulators were further analysed. m6A regulators were found to be upregulated in HCC and were significantly associated with the clinical stage of patients with HCC. In addition, higher m6A mutations were associated with shorter OS and DFS. Immune infiltration analysis showed that the YTHDF family was significantly associated with tumour immune infiltration and escape. Therefore, m6A regulators play an important role in the tumorigenesis of HCC and may be a risk factor for the survival of patients with HCC, especially the YTHDF family. Targeting the YTHDF family may be a promising uncharted area for future research into HCC treatment.

## Supplementary Information

Additional file1 (TIF 6776 KB) **Figure S1.** Prognostic, mutational and immune infiltration analysis of m6A regulators in GEO and TCGA databases. (A) Expression of m6A regulator in GEO and TCGA databases. (B) Mutation of m6A regulator in GEO and TCGA databases. (C) Immune infiltration analysis of m6A regulators in GEO and TCGA databases.

Additional file2 (TIF 16445 KB) **Figure S2. **Cut-off values for OS of m6A regulators. A cut-off plot can be used to visualize the correlation between the used cut-off values and the achieved P values (black) and hazard rate (HR) (blue). The red circle identifies the best cutoff. The computation of false discovery rate across all P values provides correction for multiple hypothesis testing.

Additional file3 (TIF 20522 KB) **Figure S3.** Prognostic feature of mRNA expression of distinct m6A regulators and PD-L1 in HCC patients (Kaplan–Meier plotter).

Additional file4 (TIF 4873 KB) **Figure S4. **Prognostic feature of mRNA expression of distinct m6A regulators in GEO and TCGA databases.

Additional file5 (TIF 12890 KB) Clinical correlation between the altered and unaltered groups of m6A regulators and PD-L1 in HCC patients. (A) Race Category; (B) Liver fibrosis ishak score category; (C) Primary Tumor Site; (D) Vascular Invasion; (E) Vascular Invasion; (F) Neoplasm Histologic Grade; (G) Neoplasm Histologic Grade; (H) Cancer Type Detailed; (I) Mutation Count; (J) Patient Height.

Additional file6 (XLSX 342 KB) **Table S1.** PCR primer sequence information.

Additional file7 (DOCX 55 KB) **Table S2. **Remarkable changes of m6A regulators expression in transcription level between HCC and normal liver tissues (ONCOMINE).

Additional file8 (DOCX 26 KB) **Table S3. **The prognostic values of m6A regulators in HCC patients (Kaplan–Meier plotter).

Additional file9 (XLSX 103 KB) **Table S4. **The related top 100 genes of m6A regulators and PD-L1.

Additional file10 (XLSX 20 KB) **Table S5. **GO and KEGG enrichment analysis of m6A regulators and their 170 co-expression genes in HCC patients.

Additional file11 (XLSX 10 KB) **Table S6. **Clinical correlation between the altered and unaltered groups of m6A regulators and PD-L1 in HCC patients.

Additional file12 (XLSX 12 KB) **Table S7. **Expression of m6A regulators and PD-L1/PDCD1 in 20 patients with hepatocellular carcinoma.

## Data Availability

The datasets generated during and/or analysed during the current study are available in the data repository: 1. ONCOMINE (http://www.oncomine.org). 2. UALCAN (http://ualcan.path.uab.edu/). 3. GEPIA (http://gepia.cancer-pku.cn/). 4. Kaplan–Meier Plotter (http://kmplot.com/analysis/). 5. TCGA Data and cBioPortal for Cancer Genomics (http://www.cbioportal.org). 6. STRING (http://www.string-db.org). 7. DAVID (https://david.ncifcrf.gov/summary.jsp). 8. TIMER2 (http://timer.cistrome.org/). 9. GEO: (https://www.ncbi.nlm.nih.gov/geo/).
