# Supplementary figures and images for: Systematic analysis of the expression profile and prognostic significance of m6A regulators and PD-L1 in hepatocellular carcinoma
Source: Discov Oncol. 2022 Nov 25;13:131. doi: 10.1007/s12672-022-00595-x (PMC9700556; doi:10.1007/s12672-022-00595-x)

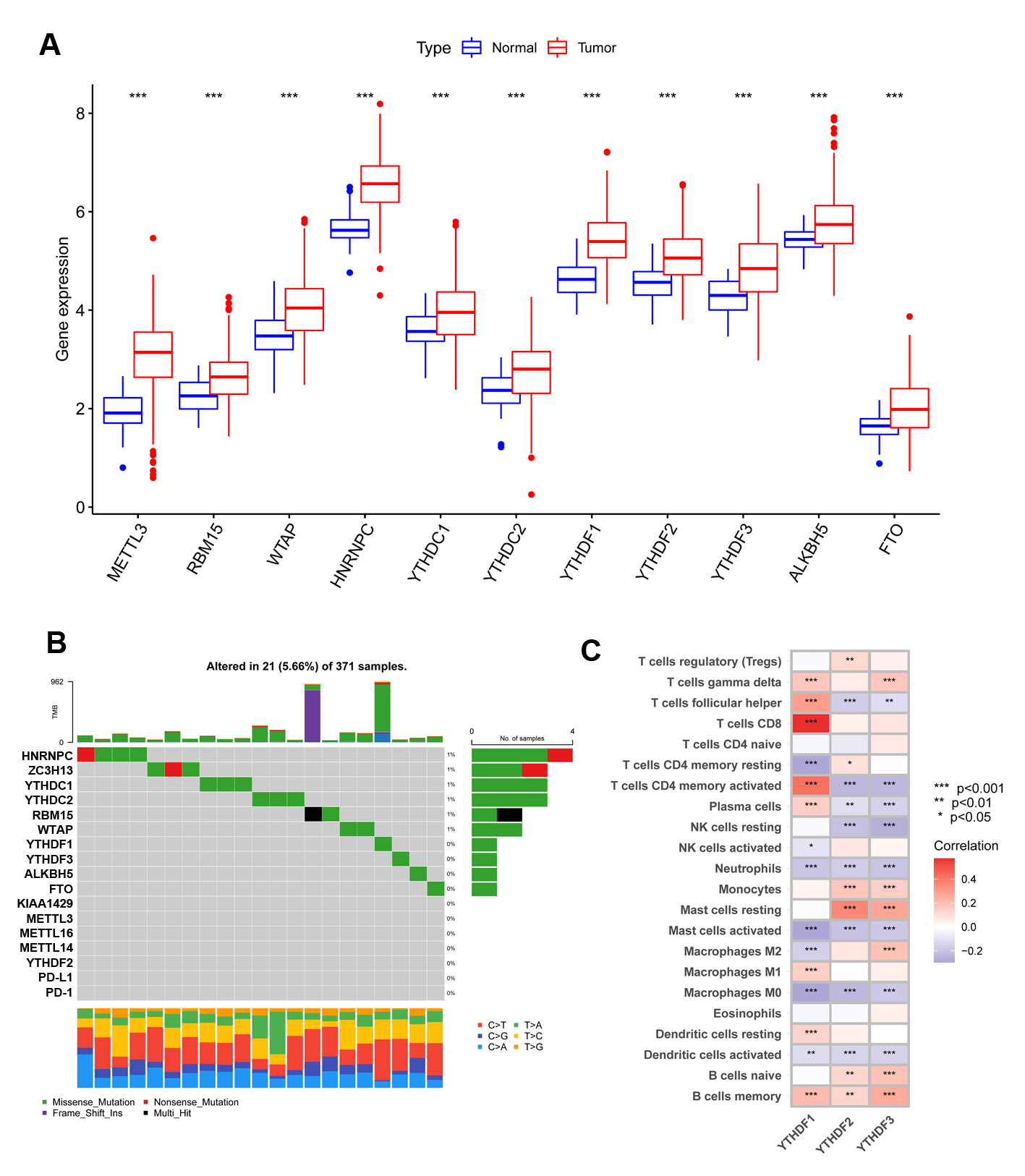

Supplement: Supplementary file 1 — Additional file1 (TIF 6776 KB) Figure S1. Prognostic, mutational and immune infiltration analysis of m6A regulators in GEO and TCGA databases. (A) Expression of m6A regulator in GEO and TCGA databases. (B) Mutation of m6A regulator in GEO and TCGA databases. (C) Immune infiltration analysis of m6A regulators in GEO and TCGA databases. [file 12672_2022_595_MOESM1_ESM.tif]

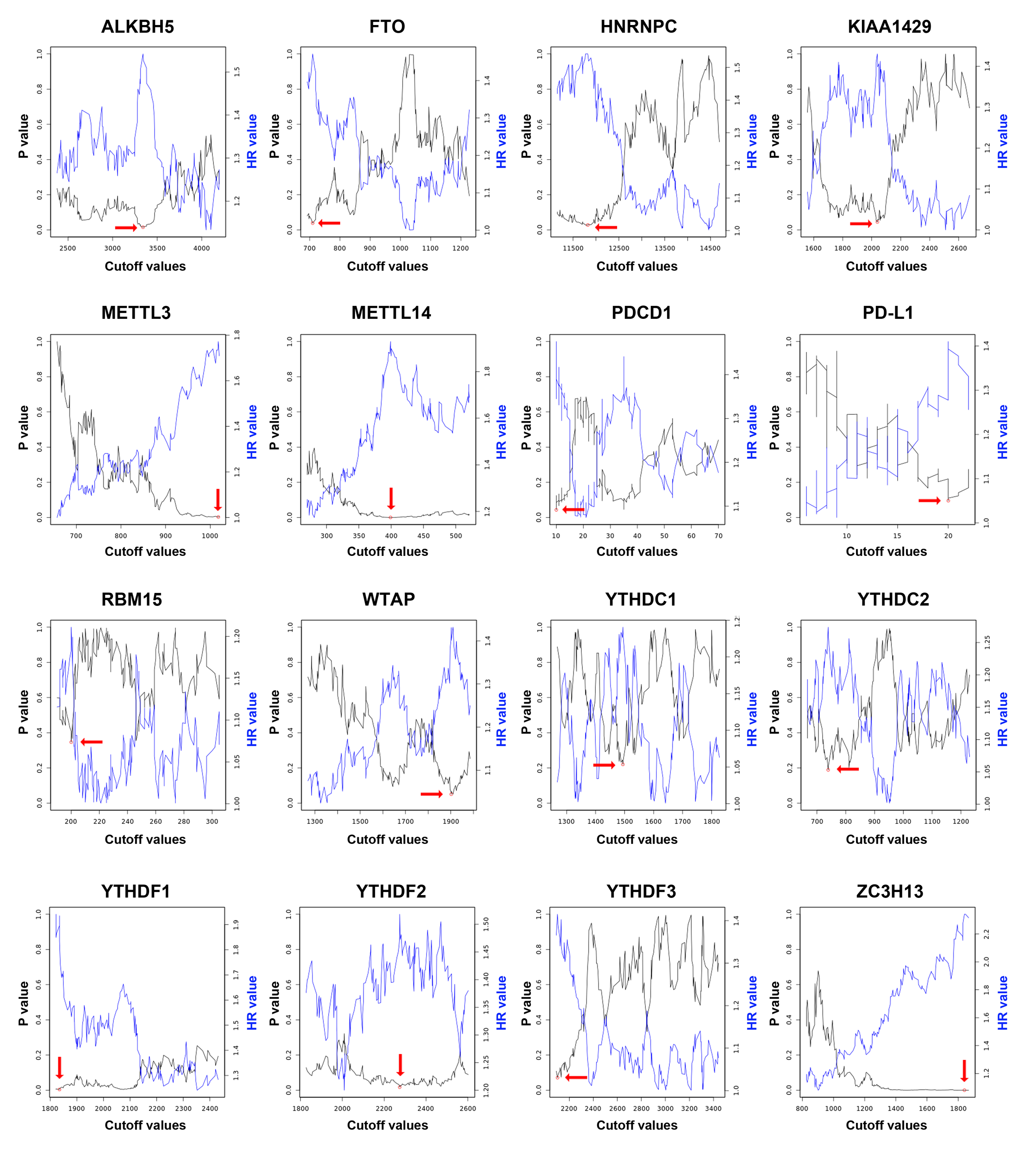

Supplement: Supplementary file 2 — Additional file2 (TIF 16445 KB) Figure S2. Cut-off values for OS of m6A regulators. A cut-off plot can be used to visualize the correlation between the used cut-off values and the achieved P values (black) and hazard rate (HR) (blue). The red circle identifies the best cutoff. The computation of false discovery rate across all P values provides correction for multiple hypothesis testing. [file 12672_2022_595_MOESM2_ESM.tif]

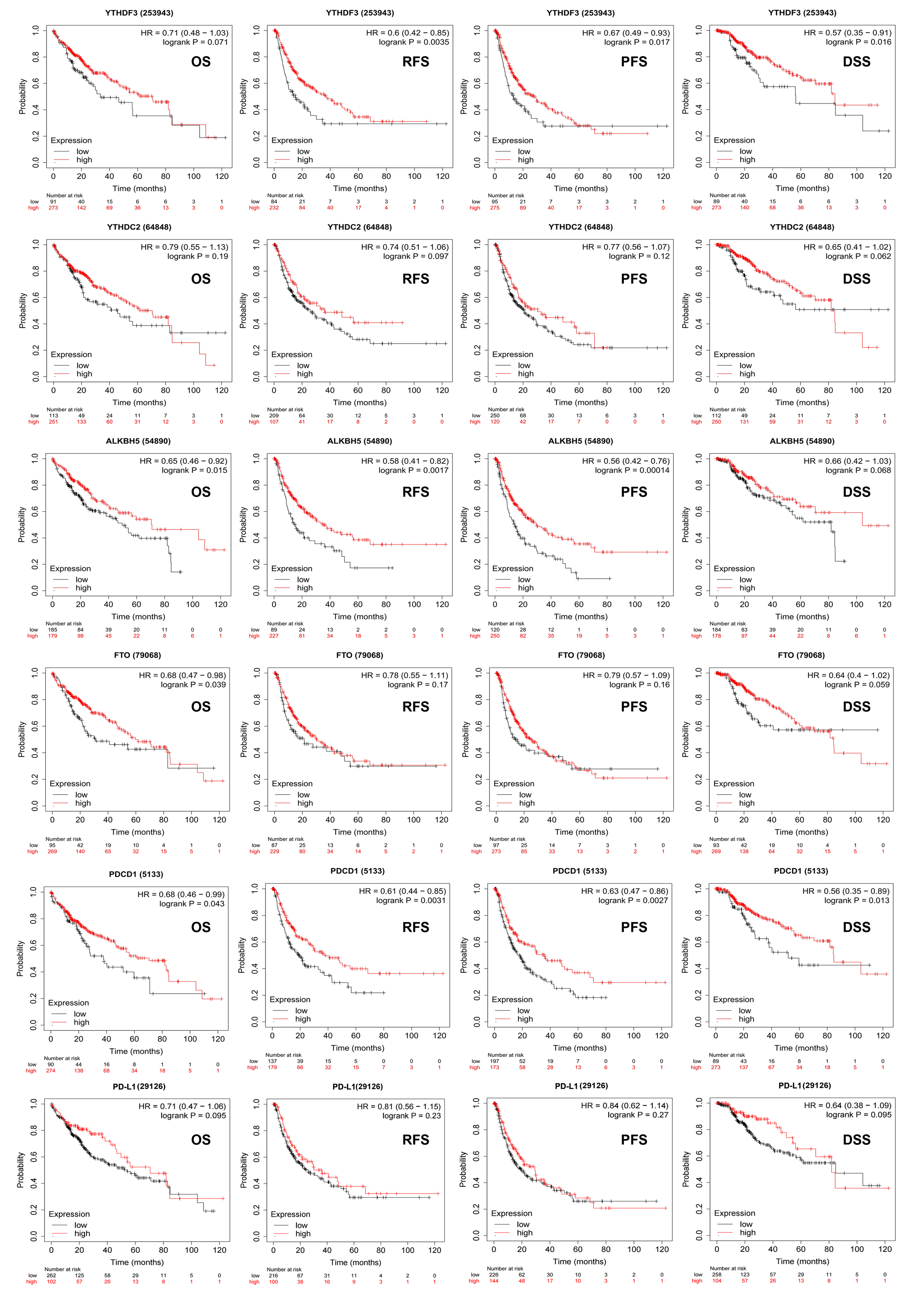

Supplement: Supplementary file 3 — Additional file3 (TIF 20522 KB) Figure S3. Prognostic feature of mRNA expression of distinct m6A regulators and PD-L1 in HCC patients (Kaplan–Meier plotter). [file 12672_2022_595_MOESM3_ESM.tif]

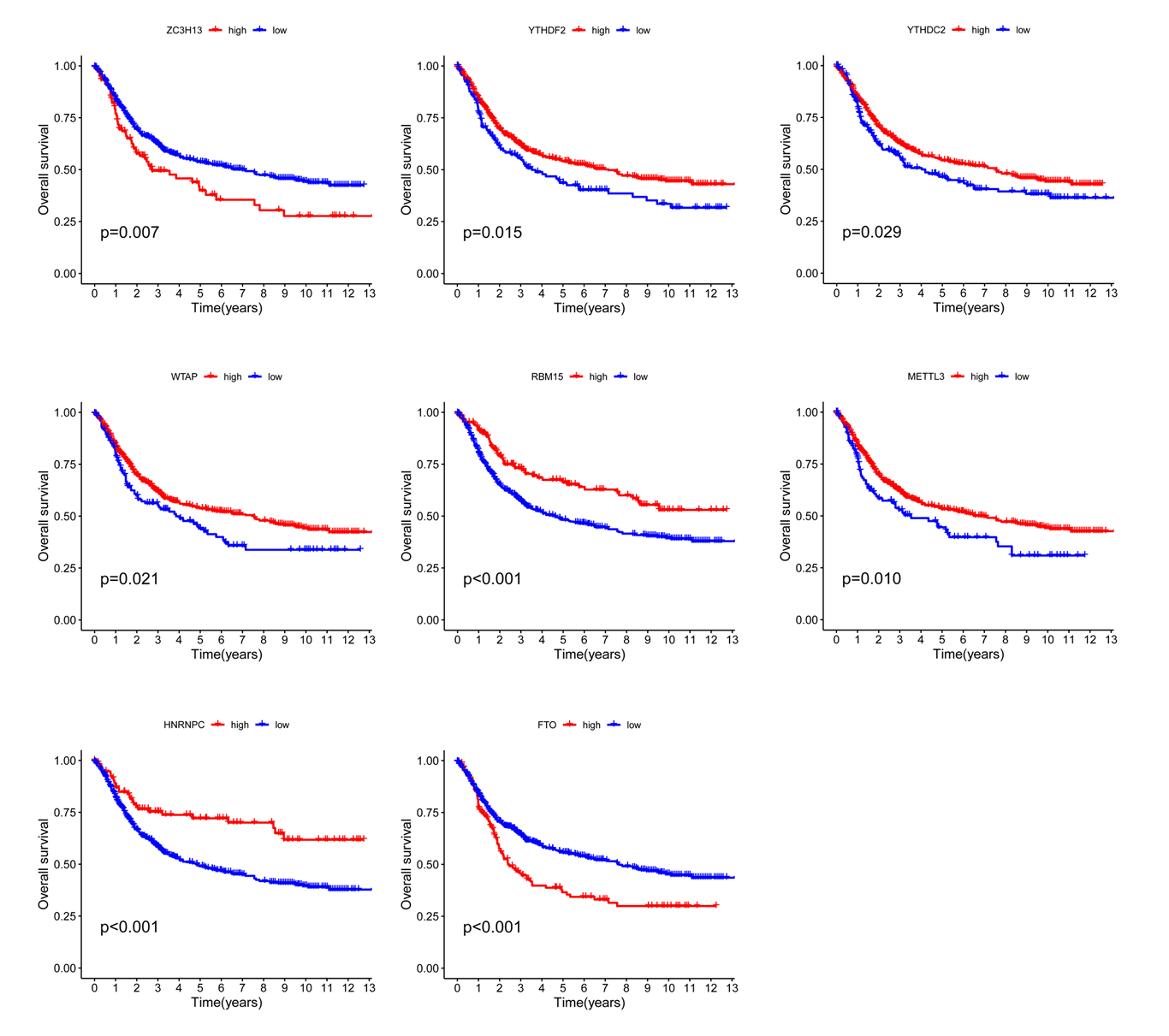

Supplement: Supplementary file 4 — Additional file4 (TIF 4873 KB) Figure S4. Prognostic feature of mRNA expression of distinct m6A regulators in GEO and TCGA databases. [file 12672_2022_595_MOESM4_ESM.tif]

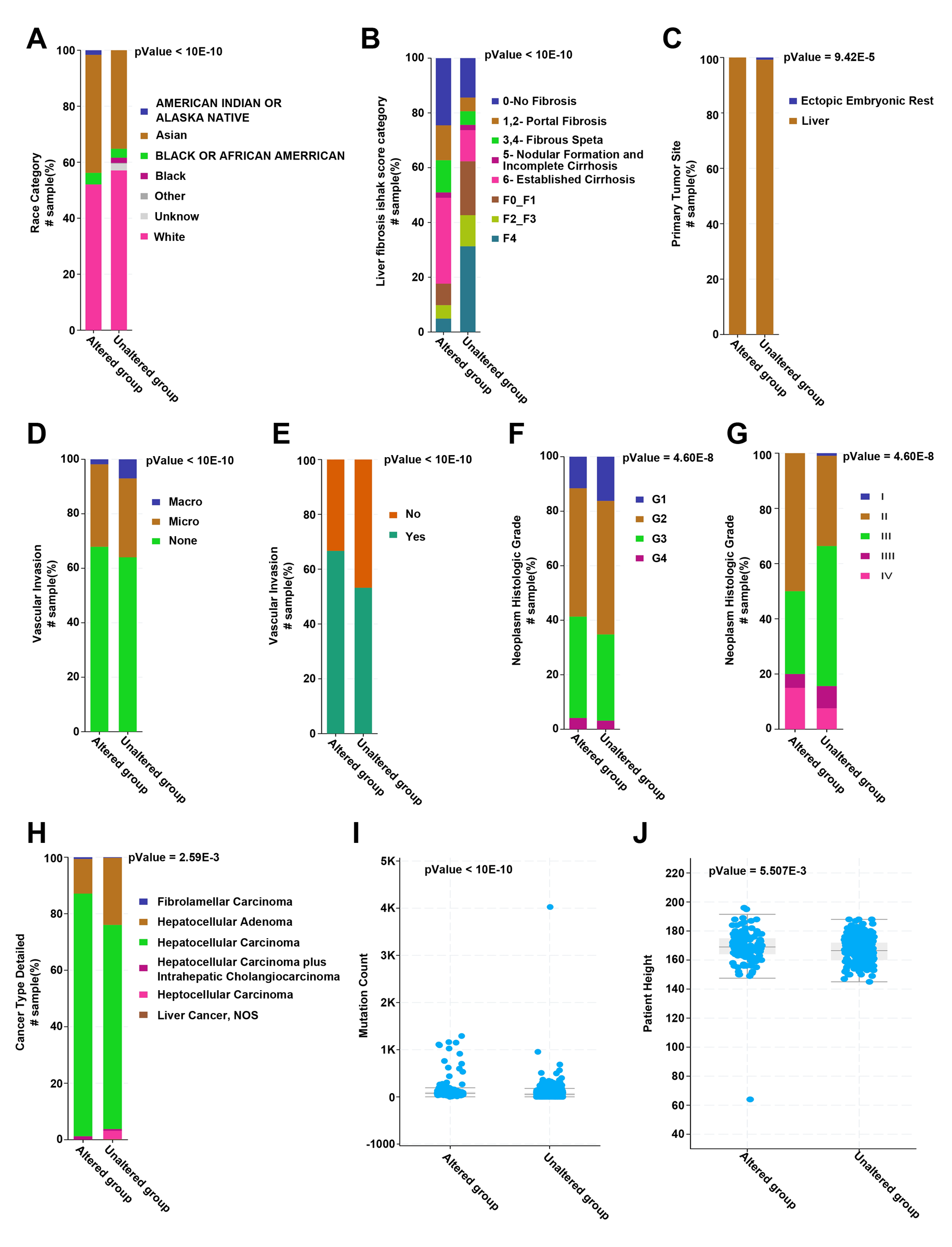

Supplement: Supplementary file 5 — Additional file5 (TIF 12890 KB) Clinical correlation between the altered and unaltered groups of m6A regulators and PD-L1 in HCC patients. (A) Race Category; (B) Liver fibrosis ishak score category; (C) Primary Tumor Site; (D) Vascular Invasion; (E) Vascular Invasion; (F) Neoplasm Histologic Grade; (G) Neoplasm Histologic Grade; (H) Cancer Type Detailed; (I) Mutation Count; (J) Patient Height. [file 12672_2022_595_MOESM5_ESM.tif]
