# Supplementary material for: Systematic analysis of the expression profile and prognostic significance of m6A regulators and PD-L1 in hepatocellular carcinoma
Source: Discov Oncol. 2022 Nov 25;13:131. doi: 10.1007/s12672-022-00595-x (PMC9700556; doi:10.1007/s12672-022-00595-x)
Supplement: Supplementary file 7 — Additional file7 (DOCX 55 KB) Table S2. Remarkable changes of m6A regulators expression in transcription level between HCC and normal liver tissues (ONCOMINE). [file 12672_2022_595_MOESM7_ESM.docx]

Table S2: Remarkable changes of m^6^A RNA methylation regulators expression in transcription level between HCC and normal liver tissues (ONCOMINE).

|  | Types of HCC vs. liver | Fold change | p-Value | t-test | References |
| --- | --- | --- | --- | --- | --- |
| KIAA1429 | Hepatocellular Carcinoma vs. Normal  Hepatocellular Carcinoma vs. Normal  Hepatocellular Carcinoma vs. Normal  Hepatocellular Carcinoma vs. Normal | 2.117  1.121  1.235  1.056 | 1.17E-9  6.62E-13  7.86E-13  2.88E-4 | 7.522  8.092  8.121  3.923 | [1]  [2]  [3]  [2] |
| METTL3 | Hepatocellular Carcinoma vs. Normal  Hepatocellular Carcinoma vs. Normal  Hepatocellular Carcinoma vs. Normal  Hepatocellular Carcinoma vs. Normal | 1.734  1.590  1.449  3.475 | 1.37E-5  4.04E-37  6.88E-5  7.92E-4 | 5.667  13.946  4.391  3.475 | [1]  [4]  [4]  [5] |
| RBM15 | Liver Cancer vs. Normal  Hepatocellular Carcinoma vs. Normal  Hepatocellular Carcinoma vs. Normal  Liver Cancer vs. Normal  Hepatocellular Carcinoma vs. Normal | 1.709  1.451  1.963  1.437  1.324 | 7.82E-4  3.56E-5  1.56E-5  0.003  4.53E-17 | 4.479  4.607  4.500  3.413  8.681 | [6]  [1]  [1]  [7]  [4] |
| WTAP | Hepatocellular Carcinoma vs. Normal | 1.420 | 2.81E-17 | 8.763 | [4] |
| HNRNPC | Hepatocellular Carcinoma vs. Normal  Hepatocellular Carcinoma vs. Normal  Hepatocellular Carcinoma vs. Normal  Hepatocellular Carcinoma vs. Normal  Hepatocellular Carcinoma vs. Normal | 1.551  1.485  1.365  1.337  1.132 | 5.44E-9  5.50E-57  2.00E-4  0.004  0.014 | 7.650  18.541  4.380  2.793  2.206 | [4]  [4]  [1]  [5]  [8] |
| YTHDC1 | Hepatocellular Carcinoma vs. Normal  Hepatocellular Carcinoma vs. Normal  Hepatocellular Carcinoma vs. Normal | 1.888  1.299  1.126 | 5.48E-20  1.62E-4  0.028 | 10.315  4.313  1.954 | [8]  [1]  [5] |
| YTHDF1 | Hepatocellular Carcinoma vs. Normal  Hepatocellular Carcinoma vs. Normal  Hepatocellular Carcinoma vs. Normal | 1.044  1.091  1.026 | 3.63E-5  4.70E-10  0.004 | 4.500  6.776  2.668 | [2]  [3]  [2] |
| YTHDF2 | Hepatocellular Carcinoma vs. Normal  Hepatocellular Carcinoma vs. Normal  Hepatocellular Carcinoma vs. Normal | 1.440  1.295  1.251 | 0.002  6.49E-13  0.019 | 3.639  7.305  2.165 | [1]  [4]  [4] |
| YTHDF3 | Hepatocellular Carcinoma vs. Normal  Hepatocellular Carcinoma vs. Normal  Hepatocellular Carcinoma vs. Normal  Hepatocellular Carcinoma vs. Normal | 1.623  1.106  1.176  1.341 | 7.61E-8  9.24E-11  3.45E-9  4.72E-17 | 6.710  7.092  6.357  8.678 | [1]  [2]  [3]  [4] |
| YTHDC2 | Hepatocellular Carcinoma vs. Normal  Hepatocellular Carcinoma vs. Normal  Hepatocellular Carcinoma vs. Normal  Hepatocellular Carcinoma vs. Normal | 1.103  1.022  1.170  1.038 | 4.71E-4  0.023  0.028  0.015 | 3.549  2.028  1.982  2.214 | [5]  [2]  [1]  [3] |
| ALKBH5 | Hepatocellular Carcinoma vs. Normal | 1.171 | 0.009 | 2.410 | [8] |

**References:**

1. Wurmbach E, Chen YB, Khitrov G, Zhang W, Roayaie S, Schwartz M, et al. Genome-wide molecular profiles of HCV-induced dysplasia and hepatocellular carcinoma. Hepatology. 2007;45(4):938-47. Epub 2007/03/30. doi: 10.1002/hep.21622.

2. Guichard C, Amaddeo G, Imbeaud S, Ladeiro Y, Pelletier L, Maad IB, et al. Integrated analysis of somatic mutations and focal copy-number changes identifies key genes and pathways in hepatocellular carcinoma. Nat Genet. 2012;44(6):694-8. Epub 2012/05/09. doi: 10.1038/ng.2256.

3. TCGA. The Cancer Genome Atlas - Hepatocellular Carcinoma DNA Copy Number Data. <http://tcga-datancinihgov/tcga/>. 2012.

4. Roessler S, Jia HL, Budhu A, Forgues M, Ye QH, Lee JS, et al. A unique metastasis gene signature enables prediction of tumor relapse in early-stage hepatocellular carcinoma patients. Cancer Res. 2010;70(24):10202-12. Epub 2010/12/17. doi: 10.1158/0008-5472.can-10-2607.

5. Mas VR, Maluf DG, Archer KJ, Yanek K, Kong X, Kulik L, et al. Genes involved in viral carcinogenesis and tumor initiation in hepatitis C virus-induced hepatocellular carcinoma. Mol Med. 2009;15(3-4):85-94. Epub 2008/12/23. doi: 10.2119/molmed.2008.00110.

6. Yu K, Ganesan K, Tan LK, Laban M, Wu J, Zhao XD, et al. A precisely regulated gene expression cassette potently modulates metastasis and survival in multiple solid cancers. PLoS Genet. 2008;4(7):e1000129. Epub 2008/07/19. doi: 10.1371/journal.pgen.1000129.

7. M B. International Genomics Consortium Expression Project for Oncology (expO) - All samples <http://wwwncbinlmnihgov/geo/query/acccgi?acc=GSE2109>.

8. Chen X, Cheung ST, So S, Fan ST, Barry C, Higgins J, et al. Gene expression patterns in human liver cancers. Mol Biol Cell. 2002;13(6):1929-39. Epub 2002/06/12. doi: 10.1091/mbc.02-02-0023.
