# Supplementary material for: Systematic analysis of the expression profile and prognostic significance of m6A regulators and PD-L1 in hepatocellular carcinoma
Source: Discov Oncol. 2022 Nov 25;13:131. doi: 10.1007/s12672-022-00595-x (PMC9700556; doi:10.1007/s12672-022-00595-x)
Supplement: Supplementary file 8 — Additional file8 (DOCX 26 KB) Table S3. The prognostic values of m6A regulators in HCC patients (Kaplan–Meier plotter). [file 12672_2022_595_MOESM8_ESM.docx]

Table S3: The prognostic values of m^6^A RNA methylation regulators in HCC patients (Kaplan–Meier plotter)

| m6A  regulators | OS | | | | RFS | | | | PFS | | | | DSS | | | |
| --- | --- | --- | --- | --- | --- | --- | --- | --- | --- | --- | --- | --- | --- | --- | --- | --- |
|  | **Cases** | **HR** | **95%CI** | ***p*-value** | **Cases** | **HR** | **95%CI** | ***p*-value** | **Cases** | **HR** | **95%CI** | ***p*-value** | **Cases** | **HR** | **95%CI** | ***p*-value** |
| KIAA1429 | **364** | **1.43** | **1.01-2.03** | **0.045** | 316 | 1.4 | 0.97-2.03 | 0.07 | 370 | 1.26 | 0.93-1.69 | 0.13 | 362 | 1.44 | 0.9-2.31 | 0.12 |
| METTL3 | **364** | **1.77** | **1.21-2.6** | **0.003** | **316** | **1.79** | **1.25-2.57** | **0.0014** | **370** | **1.57** | **1.13-2.18** | **0.0072** | **362** | **1.95** | **1.19-3.18** | **0.0068** |
| RBM15 | 364 | 1.21 | 0.81-1.81 | 0.35 | **316** | **1.41** | **0.96-2.08** | **0.0075** | **370** | **1.48** | **1.04-2.1** | **0.027** | 362 | 0.7 | 0.45-1.09 | 0.11 |
| WTAP | 364 | 1.43 | 1-2.05 | 0.051 | **316** | **1.55** | **1.11-2.17** | **0.0092** | **370** | **1.52** | **1.14-2.04** | **0.0047** | 362 | 1.44 | 0.9-2.29 | 0.12 |
| ZC3H13 | **364** | **0.43** | **0.26-0.69** | **0.00033** | **316** | **0.65** | **0.45-0.95** | **0.025** | **370** | **0.69** | **0.5-0.94** | **0.019** | **362** | **0.42** | **0.25-0.71** | **0.00082** |
| METTL14 | **364** | **0.52** | **0.36-0.75** | **0.00039** | **316** | **0.68** | **0.49-0.95** | **0.021** | 370 | 0.79 | 0.58-1.06 | 0.11 | **362** | **0.51** | **0.32-0.81** | **0.0039** |
| HNRNPC | **364** | **1.54** | **1.05-2.27** | **0.028** | 316 | 1.35 | 0.92-1.98 | 0.12 | 370 | 1.2 | 0.89-1.61 | 0.23 | **362** | **1.84** | **1.13-2.99** | **0.012** |
| YTHDC1 | 364 | 0.81 | 0.57-1.14 | 0.22 | 316 | 1.31 | 0.93-1.85 | 0.12 | **370** | **1.38** | **1.01-1.87** | **0.04** | **362** | **0.64** | **0.41-1** | **0.047** |
| YTHDF1 | **364** | **1.94** | **1.22-3.06** | **0.0042** | **316** | **1.91** | **1.36-2.66** | **0.00012** | **370** | **1.62** | **1.19-2.19** | **0.0017** | **362** | **1.85** | **1.17-2.92** | **0.0072** |
| YTHDF2 | **364** | **1.52** | **1.07-2.15** | **0.017** | **316** | **1.5** | **1.08-2.09** | **0.015** | 370 | 1.29 | 0.96-1.73 | 0.093 | 362 | 1.45 | 0.92-2.29 | 0.11 |
| YTHDF3 | 364 | 0.71 | 0.48-1.03 | 0.071 | **316** | **0.6** | **0.42-0.85** | **0.0035** | **370** | **0.67** | **0.49-0.93** | **0.017** | **362** | **0.57** | **0.35-0.91** | **0.016** |
| YTHDC2 | 364 | 0.79 | 0.55-1.13 | 0.19 | 316 | 0.74 | 0.51-1.06 | 0.097 | 370 | 0.77 | 0.56-1.07 | 0.12 | 362 | 0.65 | 0.41-1.02 | 0.062 |
| ALKBH5 | **364** | **0.65** | **0.46-0.92** | **0.015** | **316** | **0.58** | **0.41-0.82** | **0.0017** | **370** | **0.56** | **0.42-0.76** | **0.00014** | 362 | 0.66 | 0.42-1.03 | 0.068 |
| FTO | **364** | **0.68** | **0.47-0.98** | **0.039** | 316 | 0.78 | 0.55-1.11 | 0.17 | 370 | 0.79 | 0.57-1.09 | 0.16 | 362 | 0.64 | 0.4-1.02 | 0.059 |
| CD274 | 364 | 0.71 | 0.47-1.06 | 0.095 | 316 | 0.81 | 0.56-1.15 | 0.23 | 370 | 0.84 | 0.62-1.14 | 0.27 | 362 | 0.64 | 0.38-1.09 | 0.095 |
| PDCD1 | **364** | **0.68** | **0.46-0.99** | **0.043** | **316** | **0.61** | **0.44-0.85** | **0.0031** | **370** | **0.63** | **0.48-0.86** | **0.0027** | **362** | **0.56** | **0.35-0.89** | **0.013** |

(Red value mean *P*<0.05)
